# Co-Exposure to Aristolochic Acids I and II Increases DNA Adduct Formation Responsible for Aristolochic Acid I-Mediated Carcinogenicity in Rats

**DOI:** 10.3390/ijms221910479

**Published:** 2021-09-28

**Authors:** František Bárta, Alena Dedíková, Michaela Bebová, Šárka Dušková, Jaroslav Mráz, Heinz H. Schmeiser, Volker M. Arlt, Petr Hodek, Marie Stiborová

**Affiliations:** 1 Department of Biochemistry, Faculty of Science, Charles University, Albertov 2030, 128 40 Prague 2, Czech Republic; frantisek.barta@natur.cuni.cz (F.B.); hudecova@natur.cuni.cz (A.D.); m.bebova@gmail.com (M.B.); petr.hodek@natur.cuni.cz (P.H.); stiborov@natur.cuni.cz (M.S.); 2 Centre of Occupational Health, National Institute of Public Health, Šrobárova 48, 100 42 Prague 10, Czech Republic; sarka.duskova@szu.cz (Š.D.); jaroslav.mraz@szu.cz (J.M.); 3 Division of Radiopharmaceutical Chemistry, German Cancer Research Center (DKFZ), Im Neuenheimer Feld 280, 69120 Heidelberg, Germany; heinz.schmeiser1@outlook.de; 4 Department of Analytical, Environmental and Forensic Sciences Division, King’s College London, 150 Stamford Street, London SE1 9NH, UK; 5 Toxicology Department, GAB Consulting GmbH, Heinrich-Fuchs-Str. 96, 69126 Heidelberg, Germany

**Keywords:** aristolochic acid I, aristolochic acid II, aristolochic acid nephropathy, Balkan endemic nephropathy, DNA adducts, aristolochic acid-mediated carcinogenesis, genotoxicity, cytochrome P450, NAD(P)H:quinone oxidoreductase 1

## Abstract

The plant extract aristolochic acid (AA), containing aristolochic acids I (AAI) and II (AAII) as major components, causes aristolochic acid nephropathy (AAN) and Balkan endemic nephropathy (BEN), unique renal diseases associated with upper urothelial cancer. Recently (Chemical Research in Toxicology 33(11), 2804–2818, 2020), we showed that the *in vivo* metabolism of AAI and AAII in Wistar rats is influenced by their co-exposure (i.e., AAI/AAII mixture). Using the same rat model, we investigated how exposure to the AAI/AAII mixture can influence AAI and AAII DNA adduct formation (i.e., AA-mediated genotoxicity). Using ^32^P-postlabelling, we found that AA-DNA adduct formation was increased in the livers and kidneys of rats treated with AAI/AAII mixture compared to rats treated with AAI or AAII alone. Measuring the activity of enzymes involved in AA metabolism, we showed that enhanced AA-DNA adduct formation might be caused partially by both decreased AAI detoxification as a result of hepatic CYP2C11 inhibition during treatment with AAI/AAII mixture and by hepatic or renal NQO1 induction, the key enzyme predominantly activating AA to DNA adducts. Moreover, our results indicate that AAII might act as an inhibitor of AAI detoxification *in vivo*. Consequently, higher amounts of AAI might remain in liver and kidney tissues, which can be reductively activated, resulting in enhanced AAI DNA adduct formation. Collectively, these results indicate that AAII present in the plant extract AA enhances the genotoxic properties of AAI (i.e., AAI DNA adduct formation). As patients suffering from AAN and BEN are always exposed to the plant extract (i.e., AAI/AAII mixture), our findings are crucial to better understanding host factors critical for AAN- and BEN-associated urothelial malignancy.

## 1. Introduction

**Figure 1:**
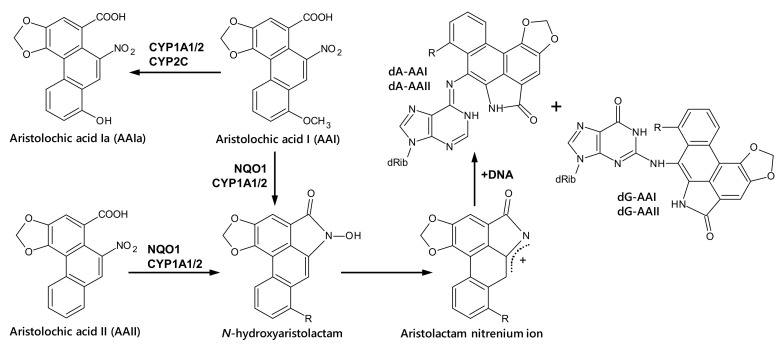
Pathways of bioactivation and DNA adduct formation by AAI and AAII: 7-(deoxyadenosin-*N*^6^-yl)-aristolactam I (dA-AAI); 7-(deoxyguanosin-*N*^2^-yl)-aristolactam I (dG-AAI); 7-(deoxyadenosin-*N*^6^-yl)-aristolactam II (dA-AAII); 7-(deoxyguanosin-*N*^2^-yl)-aristolactam II (dG-AAII). See text for details.

Aristolochic acid (AA) is the natural plant extract of both the *Aristolochia* and *Asarum* genera of the family *Aristolochiaceae*, namely *Aristolochia clematitis* in particular in Europe [1]. The plant extract consists of structurally related nitrophenanthrene carboxylic acids, with aristolochic acid I (8-methoxy-6-nitro-phenanthro-(3,4-*d*)-1,3-dioxolo-5-carboxylic acid, AAI) and aristolochic acid II (6-nitro-phenanthro-(3,4-*d*)-1,3-dioxolo-5-carboxylic acid, AAII) being the major components (Figure 1). AAI and AAII are both mutagenic and genotoxic compounds [2,3,4,5,6,7]. In 2012, AA was classified as carcinogenic to humans (group 1) by the International Agency for Research on Cancer (IARC), acting via a genotoxic mechanism [8]. Today, there is overwhelming evidence that human exposure to AA leads to chronic renal disease and upper urothelial cancer (UUC), known as aristolochic acid nephropathy (AAN) [8,9,10], which is now recognised as a global disease [10,11]. Further, AA is also considered to be the cause of another chronic renal disease associated with urothelial malignancy known as Balkan endemic nephropathy (BEN) [12,13,14,15,16,17].

AAI is considered to be responsible for AA-mediated nephropathy by directly causing interstitial renal injury [18]; however, enzymatic activation of AAI to intermediates capable of binding to DNA is a necessary reaction leading to AA-mediated genotoxicity and malignant transformation [12,13,14,19,20,21,22] (Figure 1). Genotoxicity results not only from enzymatic bioactivation of AAI but also from AAII [23,24,25,26,27,28,29,30]. Initial nitro reduction of AAI and AAII to *N*-hydroxyaristolactam I (*N*-OH-Alac I) and *N*-hydroxyaristolactam II (*N*-OH-Alac II), respectively, is required to exert their genotoxic properties. *N*-OH-Alac I and II generate cyclic acylnitrenium ions capable of binding to DNA, preferentially forming pre-mutagenic purine DNA adducts [23,24,31,32]. In the target tissues of AAN AND BEN patients, 7-(deoxyadenosin-*N*^6^-yl)aristolactam I (dA-AAI) is the most abundant DNA adduct detected [2,16,31,32,33,34,35,36,37]. This adduct also shows a long persistence in renal tissue of the patients and is still detectable decades after AA exposure [32]. It causes characteristic A:T→T:A transversion mutations, which have been found in high frequency in the whole genome of urothelial tumours of AAN AND BEN patients, including critical genes of carcinogenesis, such as the tumour suppressor gene *TP53* [4,13,15,38,39,40,41,42].

Comparing DNA adduct formation with AAI and AAII *in vivo*, significantly higher adduct levels were detected in several organs of rats and mice when treated with AAI than with AAII [23,25,43,44]. Similar results were found in various enzymatic systems *in vitro* [14,26,27,28,30,45]. These differences in the levels of AAI- and AAII-derived DNA adducts found *in vivo* and *in vitro* might be caused by differences in the enzymatic conversion of both compounds, which lead to their activation (i.e., DNA adduct formation) and detoxification.

Although the plant extract AA and its main components AAI and AAII are classified as group 1 human carcinogens by IARC, there are still important questions which need to be addressed to fully understand the underlying mechanisms involved in the development of AAN AND BEN. One key question is how AAI and AAII contribute to the toxicity (nephrotoxicity) or genotoxicity (carcinogenicity) of the plant extract, since only a small proportion of AA-exposed individuals develop AAN/BEN and associated UUC. In this context, it is noteworthy that the contents of AAs and their derivatives vary widely in *Aristolochia species* growing both in Europe (Mediterranean) and Asia [1,46,47]. This may cause different levels of (geno)toxicity in the individuals exposed to the plant extract AA. Some *Aristolochia* plants contain more AAI, while in others AAII prevails, with amounts of AAI and AAII ranging between 40 and 60% [1,47]. Consequently, the ratio of AAI to AAII in the natural plant extract AA can impact on the results when tested *in vitro* and *in vivo*; thus, investigating whether and how the ratios of AAI and AAII in plants influence the *in vivo* metabolism of AAI and AAII and subsequently the formation of AAI- and AAII-derived DNA adducts can help answer this question. 

In this experimental model, rats were previously exposed to AAI, AAII and an AAI/AAII mixture [48]. As part of a complex investigation, the first results showed that the *in vivo* metabolism of AAI and AAII in rats is influenced by the presence of both AAs. Exposure to the AAI/AAII mixture affected the generation of their urinary metabolites formed by oxidation, reduction and conjugation reactions [48]. For instance, the reductive (activation) metabolism of AAI was increased in the presence of AAII, while in the presence of AAI the reductive metabolism of AAII decreased. These results suggested that increased AAI bioactivation in the presence of AAII might also lead to increased AAI genotoxicity, which critically impacts on AAI-mediated carcinogenesis; therefore, in the present study, we investigated how exposure to the AAI/AAII mixture can influence AA-mediated genotoxicity (i.e., formation of AAI and AAII DNA adducts). For these reasons, we analysed the formation of AAI- and AAII-derived DNA adducts in liver and kidney tissues of rats treated with AAI, AAII or the AAI/AAII mixture by ^32^P-postlabelling. In addition, since AA metabolism dictates the levels of AA-DNA adducts formed, we also examined the activity of enzymes involved in AAI/AAII bioactivation and detoxification and how enzyme activity levels are influenced when rats are exposed to AAI, AAII or the AAI/AAII mixture. Specifically, we examined the activity of NAD(P)H:quinone oxidoreductase 1 (NQO1), a key enzyme involved in the reductive activation of AA. Further, we evaluated cytochrome P450 (CYP) enzymes such as CYP1A1 and 1A2, which contribute not only to the reductive activation of AA, but also to its oxidative detoxification. The consequences of AA-mediated alternations in the activity levels of these enzymes on oxidation or reduction of AAI and AAII and subsequent AAI- and AAII-derived DNA adduct formation *in vitro* were also investigated.

## 2. Results

### 2.1. DNA Adduct Formation in Rats Treated with AAI, AAII or AAI/AAII Mixture 

**Figure 2:**
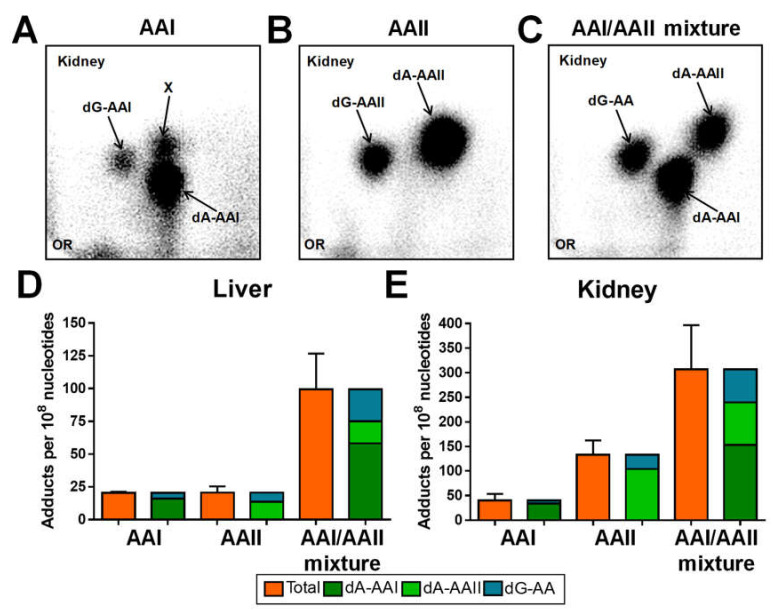
Representative autoradiograms of DNA adducts, measured by ^32^P-postlabelling, in kidney tissue samples of rats treated with AAI (**A**), AAII (**B**) or AAI/AAII mixture (**C**). These profiles are representative of adduct pattern obtained with DNA from liver tissue samples of the same rats. The origin (OR) on the TLC plate, shown in the bottom left-hand corners, was cut off before imaging. Note:: 7-(deoxyadenosin-*N*^6^-yl)-aristolactam I (dA-AAI); 7-(deoxy- guanosin-*N*^2^-yl)-aristolactam I (dG-AAI); 7-(deoxyadenosin-*N*^6^-yl)-aristolactam II (dA-AAII); 7-(deoxy- guanosin-*N*^2^-yl)-aristolactam II (dG-AAII); dG-AA, mixture of dG-AAI and dG-AAII; X, unknown adduct whose chemical structure has not yet been identified. Total DNA adduct levels measured by quantitative ^32^P-postlabelling analysis in liver (**D**) and kidney (**E**) tissues of rats treated with AAI, AAII or AAI/AAII mixture. All values are given as the means ± SD (*n* = 2). Note the different scaling levels when comparing AA-DNA adduct levels in liver and kidney tissue samples.

AAI and AAII DNA adduct formation was determined by ^32^P-postlabelling in liver and kidney samples of male Wistar rats treated i.p. with a single dose of AAI (20 mg/kg bw), AAII (20 mg/kg bw) or AAI/AAII mixture (containing each 20 mg/kg bw AAI and AAII). The adduct patterns obtained by TLC ^32^P-postlabelling were distinct for each treatment group but qualitatively similar in both rat organs. As shown in Figure 2A, the adduct pattern induced by AAI consisted of two major adduct spots previously identified [23,25] as 7-(deoxyadenosin-*N*^6^-yl)-aristolactam I (dA-AAI) and 7-(deoxy- guanosin-*N*^2^-yl)-aristolactam I (dG-AAI). Moreover, in kidney but not liver DNA samples, another adduct spot (assigned as spot X) was detectable, the chemical structure of which has not yet been elucidated. To allow comparison, adduct spot X was excluded from the quantification of total AA-DNA adduct levels. After treatment with AAII, two major adduct spots were observed, which were previously identified [24,25] as 7-(deoxyadenosin-*N*^6^-yl)-aristolactam II (dA-AAII) and 7-(deoxyguanosin-*N*^2^-yl)- aristolactam II (dG-AAII) (Figure 2B). After exposure of rats to the AAI/AAII mixture, the adduct pattern consisted of 3 major adduct spots. Two of the adduct spots were identified as dA-AAI and dA-AAII. The remaining adduct spot was assigned to dG-AA consisting of dG-AAI and dG-AAII (overlapping each other), which could not be distinctly separated on TLC under the chromatographic conditions used (Figure 2C); thus, for the AAI/AAII mixture, only the total dG-AA was quantified. Most AA-derived DNA adducts (i.e., dA-AAI, dG-AAI and dA-AAII) detected in rats have also been found in urothelial tissue of AAN/BEN patients [2,16,29,31,33,34,36]. No AA-derived DNA adducts were found in liver and kidney DNA samples of untreated (control) rats. 

Generally, AA-DNA adduct levels were higher in the kidneys, the target organs of AAI genotoxicity, than in the liver, the organ predominantly responsible for AA biotransformation (Figure 2D,E). While the total levels of DNA adducts induced by AAI and AAII in the liver were similar (~20 adducts per 10^8^ nucleotides) (Figure 2D), adduct levels induced by AAII in the kidneys were around 3-fold higher than those induced by AAI (~40 adducts per 10^8^ nucleotides for AAI *versus* ~130 adducts per 10^8^ nucleotides for AAII) (Figure 2E). In both organs treated with the AAI/AAII mixture, the total AA-DNA adduct levels were up to ~2.5-fold higher than the sum of the DNA adducts formed in rats treated with AAI and AAII individually (Figure 2D,E); however, even more importantly, looking specifically at dA-AAI and dA-AAII levels, the differences in adduct levels were even more striking. The levels of dA-AAI were up to 4.5-fold higher in the organs of rats treated with the AAI/AAII mixture than in rats exposed to AAI alone; therefore, an increase in the reductive activation of AAI in rats in the presence of AAII, which leads to the formation of dA-AAI adducts, may critically impact on AAI-mediated carcinogenesis, as this pre-mutagenic lesion causes characteristic A:T→T:A transversion mutations in urothelial tumours of AAN/BEN patients [4,15,38]. In contrast, the levels of dA-AAII practically remained unchanged in the organs of rats treated with the AAI/AAII mixture and in rats exposed to AAII alone.

### 2.2. Activity Levels of Biotransformation Enzymes in the Liver and Kidneys of Rats Treated with AAI, AAII or AAI/AAII Mixture

**Figure 3:**
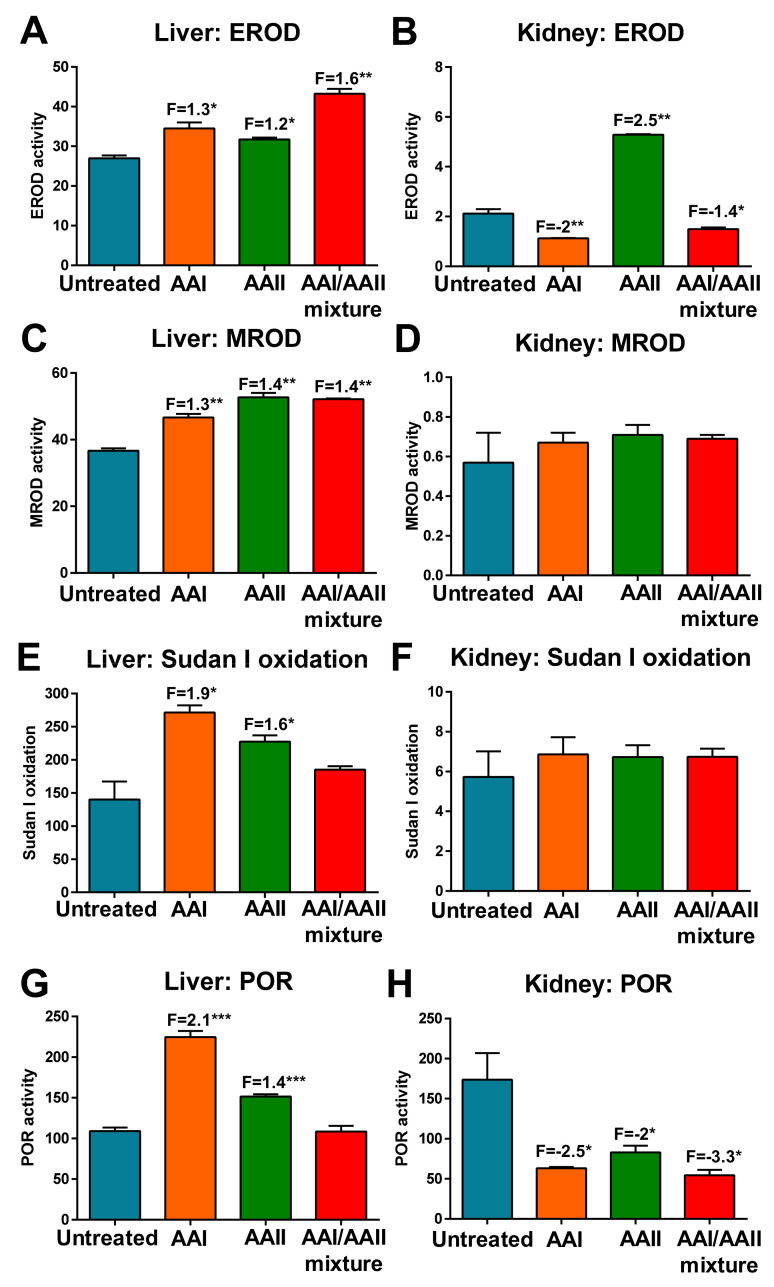

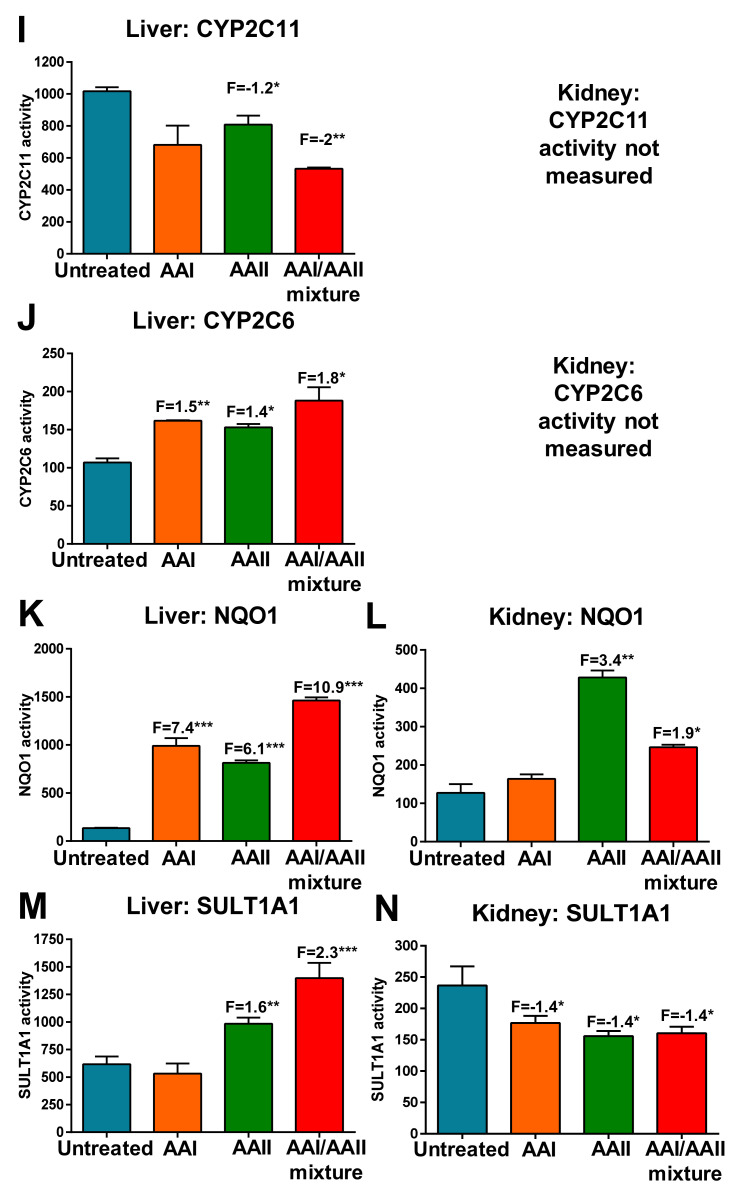
Measurement of enzyme activity levels in microsomal and cytosolic fractions of livers (left panels) and kidneys (right panels) from control (untreated) rats and rats treated with AAI, AAII, or AAI/AAII mixture. CYP1A enzymatic activity in microsomal fractions as measured by EROD activity (picomoles resorufin per minute per milligram protein) (**A**,**B**), MROD activity (picomoles resorufin per minute per milligram protein) (**C**,**D**) or Sudan I oxidation (picomole total C-hydroxylated metabolites per minute per milligram protein) (**E**,**F**). POR enzyme activity was measured as nmol of cytochrome *c*/mg/minute (**G**,**H**). CYP2C11 enzyme activity was measured as testosterone 16α-hydroxylation (picomoles 16α-hydroxytestosterone per minute per milligram protein) (**I**). CYP2C6 enzyme activity was measured as diclofenac 4′-hydroxylation (picomoles 4′-hydroxydiclofenac per minute per milligram protein) (**J**). CYP2C11 and CYP2C6 activity was only measured in hepatic microsomes. NQO1 enzyme activity was determined using menadione and cytochrome *c* as the substrate and expressed as nanomoles cytochrome *c*/minutes/mg protein (**K**,**L**). SULT1A1 enzyme activity was determined using a colorimetric assay with *p*-nitrophenol sulfate as the sulfo-donor and is expressed as picomoles *p*-nitrophenol/minute/mg protein (**M**,**N**). All values are given as the means ± SD (*n* = 3). Numbers above columns (‘F’) indicate fold changes in enzyme activity levels compared to control (untreated). Comparison was performed by *t*-test analysis: * *p* < 0.05, ** *p* < 0.01, *** *p* < 0.001, differences from control. Note different scaling when comparing enzymatic activity levels in hepatic and renal microsomal or cytosolic fractions.

Because NQO1 and CYP1A1/2 enzymes can reduce (i.e., activate) AAI and AAII, their expression might dictate the formation of AA-DNA adducts; however, as CYP1A1/2 enzymes also oxidise AAI (i.e., detoxify AAI) but not AAII [49], their expression might determine the balance between activation and detoxification pathways of AAI [14,19,20]. As such, we investigated whether the activity levels of these enzymes in the liver and kidneys is influenced by treatment of rats with AAI, AAII or AAI/AAII mixture, thereby affecting AAI and AAII DNA adduct formation *in vivo* (compare Figure 2). The activity levels of these enzymes involved in AAI and AAII biotransformation were determined using the marker substrates (reactions) (Figure 3). 

Marker activity of the CYP1A (EROD) was detectable in both organs (Figure 3A,B). In the liver, EROD activity was significantly induced in all treatment groups (1.2–1.6-fold), with the highest induction level seen in rats treated with the AAI/AAII mixture (Figure 3A). In the kidneys, EROD activity was strongly increased in rats treated with AAII, while in rats exposed to AAI, either alone or in combination with AAII (i.e., AAI/AAII mixture), the activity was actually significantly reduced (Figure 3B). MROD activity, a marker reaction for CYP1A2, was found in the liver (Figure 3C) but showed very low amounts in the kidneys (Figure 3D), confirming that CYP1A2 is almost exclusively a hepatic enzyme [50]. Treatment of rats with AAI, AAII or AAI/AAII mixture led to a significant increase in MROD activity (up to 1.4-fold) in the liver but with no difference observed between the AA treatment groups (Figure 3C). In the kidneys, AA treatment had no effect on MROD activity (Figure 3D). Similar results were obtained when CYP1A1 enzyme activity was assessed via the oxidation of Sudan I to its C-hydroxylated metabolites. In the liver, treatment with AAI (1.9-fold) and AAII (1.6-fold) led to a significant induction of Sudan I oxidation; after treatment with the AAI/AAII mixture, Sudan I oxidation was higher than in the controls (untreated) but statistical significance was not reached (Figure 3E). In the kidneys, again AA treatment had no effect on Sudan I oxidation (Figure 3F). The activity of POR was increased in the liver samples of rats exposed to AAI or AAII, but not after treatment with the AAI/AAII mixture (Figure 3G). The POR enzyme activity was decreased in kidney samples of rats who received AA treatment relative to control (untreated) rats (Figure 3H). POR not only acts as an electron donor in catalytic functions of CYPs, thereby modulating the activity of CYP enzymes, but it is also able to reductively activate AAI, and to some extent AAII [27,28,51]; however, the observed changes in POR caused by AA treatment did not influence the activity of CYP1A1/2 (compare Figure 3A–F). In rat liver samples, the major contribution to AAI oxidative detoxification to AAIa was attributed to the CYP2C subfamily, particularly CYP2C6 (~17%) and 2C11 (~42%) [52]; therefore, the effects of AAI and AAII on the specific activity of CYP2C were also investigated. The activity levels of CYP2C6 in rat liver samples were increased (up to 1.8-fold) after treatment with AAI, AAII and AAI/AAII mixture relative to control (untreated) rats (Figure 3J). Nevertheless, AAI and AAII treatment decreased the activity levels of CYP2C11 in hepatic microsomes, while the greatest decrease (2-fold) was seen after treatment with AAI/AAII mixture (Figure 3I).

Treatment of rats with AAI, AAII and AAI/AAII mixture led to increased enzyme activity of cytosolic NQO1 in both organs (Figure 3K,L). The effects of treatment of rats with AAs were always higher in cytosols of the liver than the kidneys. In liver samples, NQO1 activity was the highest after treatment with AAI/AAII mixture (Figure 3K), whereas in kidney samples, the strongest effect was seen in AAII-treated rats (Figure 3L). These findings indicate that both AAI and AAII act as strong inducers of NQO1 in rats and that the effect of hepatic NQO1 induction is further increased when both AAs are administered together. The enzyme activity of hepatic sulfotransferase 1A1 (SULT1A1) was significantly increased after treatment with AAII (1.6-fold) and AAI/AAII mixture (2.3-fold) (Figure 3M); however, no significant effect on this enzyme activity was found in hepatic cytosols of rats treated with only AAI. In kidney samples, AA treatment led to decreased (1.4-fold) SULT1A1 enzyme activity in all treatment groups (Figure 3N). 

### 2.3. AAIa Formation in Hepatic and Renal Microsomes Isolated from Rats Treated with AAI, AAII or AAI/AAII Mixture

**Figure 4:**
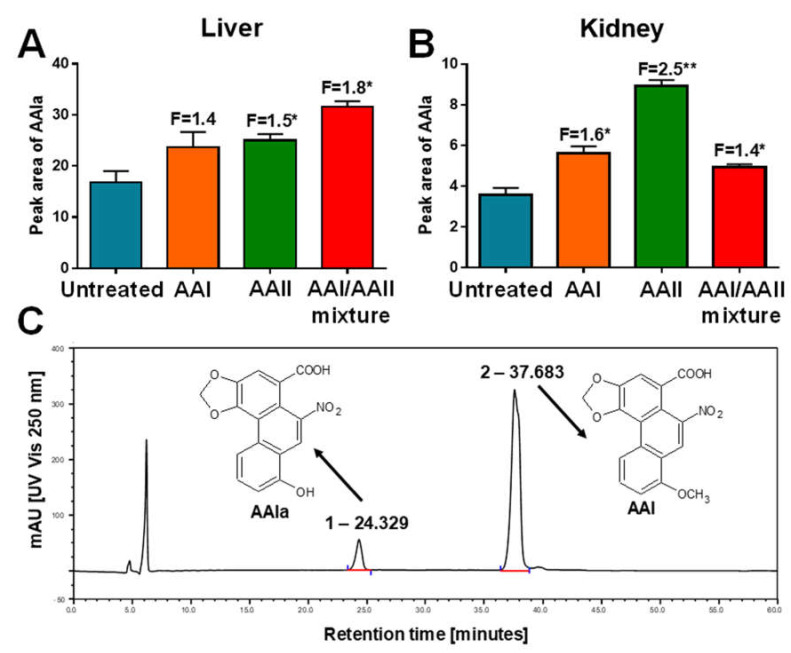
Oxidation of AAI to AAIa by microsomes of livers (**A**) and kidneys (**B**) from control (untreated) rats and rats pre-treated with AAI, AAII, or AAI/AAII mixture. All values are given as the means ± SD (*n* = 3). Numbers above columns (‘F’) indicate fold changes in AAIa levels compared to control (untreated). Comparison was performed by *t*-test analysis: * *p* < 0.05, ** *p* < 0.01, differences from control (untreated). Representative HPLC chromatographs of AAIa metabolite (peak r.t. at 24.3 min) and AAI (peak r.t. at 37.7 min) produced by rat microsomes incubated with AAI and NADPH (**C**). The peaks with the characteristic AAI metabolite (AAIa) and the parent AAI are indicated in the chromatograms.

To study AAI detoxification, incubation with AAI in the presence of hepatic or renal microsomes was carried out under aerobic conditions. All microsomes oxidised AAI to AAIa, as demonstrated by one metabolite being detectable by HPLC (peak r.t. 24.329 min) (Figure 4C). The amounts of AAIa were significantly increased in all treatment groups in both organs (Figure 4A,B). In liver samples, microsomes from rats pre-treated with AAI/AAII mixture produced the highest amount of AAIa (Figure 4A), while in kidney samples pre-treatment with AAII resulted in the highest amount of AAIa (Figure 4B). In hepatic microsomes, increased AAI demethylation after pre-treatment of rats with AAI, AAII, or AAI/AAII mixture seemed to be paralleled by increased CYP1A enzyme activity. In contrast, the results obtained in renal microsomes were complex. While the increase in AAI demethylation after pre-treatment of rats with AAII appeared to parallel increased EROD activity, decreases in EROD activity in the livers of rats treated with AAI or AAI/AAII mixture did not correlate with the increased AAIa formation observed in these animals.

### 2.4. DNA Adduct Formation by AAI and AAII Ex Vivo Involving Incubation with Hepatic and Renal Microsomal and Cytosolic Fractions Isolated from Rats Treated with AAI, AAII or AAI/AAII Mixture 

In additional experiments, we investigated the ability of microsomal and cytosolic fractions isolated from the livers and kidneys of rats treated with AAI, AAII or AAI/AAII mixture to catalyse AAI- and AAII-derived DNA adduct formation. Microsomal and cytosolic fractions from control (untreated) animals were used for comparison. NADPH-dependent DNA adduct formation after AAI and AAII incubation with cytosols and microsomes was used as a measure of AAI and AAII bioactivation by cytosolic NQO1 and microsomal CYPs, respectively. In these experiments, the ^32^P-postlabelling assay was used again to determine AA-DNA adduct formation. The adduct pattern found on the TLC sheets when using incubations of hepatic and renal microsomes and cytosols with AAI and AAII was qualitatively identical to those observed *in vivo* in liver and kidney tissues of rats treated with either AAI or AAII (compare Figure 2A,B). After incubation with AAI, the adduct pattern consisted of three adduct spots, namely dA-AAI, dG-AAI and dA-AAII. We have shown previously that the dA-AAII adduct can also be generated from AAI, probably *via* a demethoxylation reaction of AAI or dA-AAI [25,29]. For AAII, two adduct spots were detected on TLC after incubation with cytosolic and microsomal fractions, namely dA-AAII and dG-AAII. No DNA adducts were observed in control incubations carried out in parallel.

**Figure 5:**
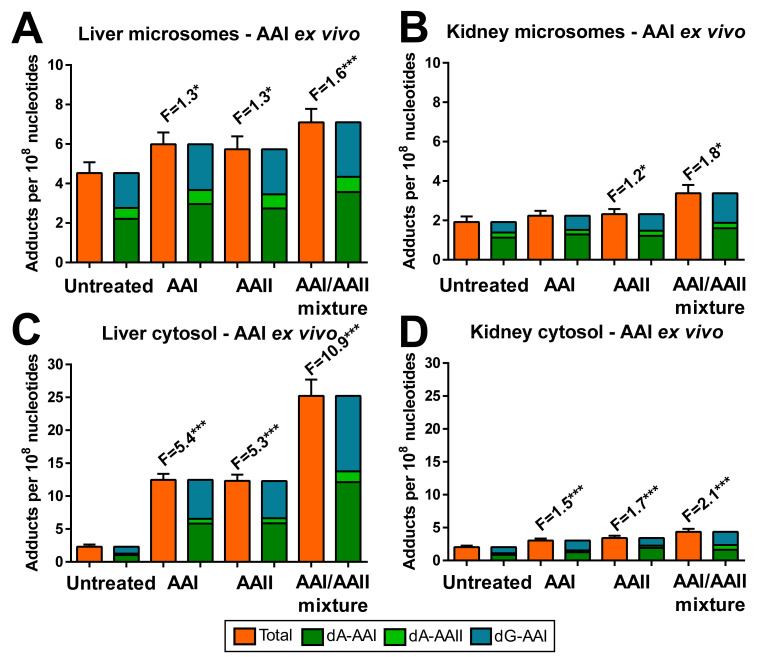
DNA adduct formation *ex vivo* by AAI in rat microsomal fractions of livers (**A**) and kidneys (**B**) from control (untreated) rats and rats pre-treated with AAI, AAII or AAI/AAII mixture, as determined by ^32^P-postlabelling. DNA adduct formation *ex vivo* by AAI in rat cytosolic fractions of livers (**C**) and kidneys (**D**) from control (untreated) and rats pre-treated with AAI, AAII or AAI/AAII mixture, as determined by ^32^P-postlabelling. All values are given as the means ± SD (*n* = 3). Numbers above columns (‘F’) indicate fold changes in DNA adduct levels compared to control (untreated). Comparison was performed by *t*-test analysis: * *p* < 0.05, *** *p* < 0.001, differences from control (untreated). Note different scaling when comparing AA-DNA adduct levels in microsomal and cytosolic fractions. Negative control incubations were performed either (*i*) without cytosol or microsomes, (*ii*) without NADPH, (*iii*) without DNA or (*iv*) without AAI and were devoid of DNA adducts.

**Figure 6:**
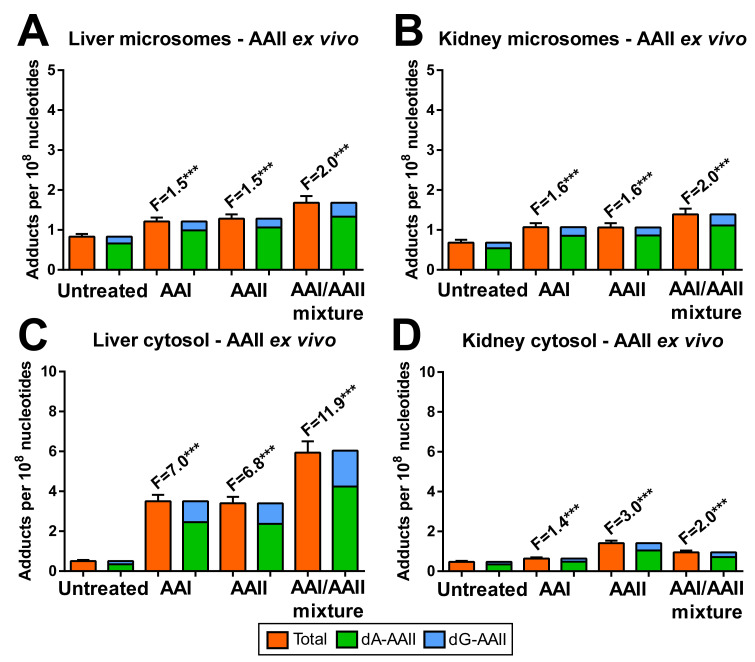
DNA adduct formation *ex vivo* by AAII in rat microsomal fractions of livers (**A**) and kidneys (**B**) from control (untreated) and rats pre-treated with AAI, AAII or AAI/AAII mixture, as determined by ^32^P-postlabelling. DNA adduct formation *ex vivo* by AAII in rat cytosolic fractions of livers (**C**) and kidneys (**D**) from control (untreated) and rats pre-treated with AAI, AAII or AAI/AAII mixture, as determined by ^32^P-postlabelling. All values are given as the means ± SD (*n* = 3). Numbers above columns (‘F’) indicate fold changes in DNA adduct levels compared to control (untreated). Comparison was performed by *t*-test analysis: *** *p* < 0.001, differences from control (untreated). Note: Different scaling was used when comparing AA-DNA adduct levels in microsomal and cytosolic fractions. Negative control incubations were performed either (*i*) without cytosol or microsomes, (*ii*) without NADPH, (*iii*) without DNA or (*iv*) without AAI and were devoid of DNA adducts.

The AAI-derived DNA adducts formed by hepatic and renal microsomes and cytosols are shown in Figure 5, while the AAII-derived DNA adducts are shown in Figure 6. Total AA-DNA adduct levels ranged from ~0.5 to 25 adducts per 10^8^ nucleotides, with levels being up to ~5-fold higher for DNA adducts generated by incubation with AAI than AAII. Pre-treatment of rats with AAI, AAII or AAI/AAII mixture increased the levels of AAI- and AAII-derived DNA adducts when AAI and AAII were incubated with DNA and cytosolic subcellular fractions *ex vivo*. Increased AA-DNA adduct levels correlated with NQO1 enzyme activity in cytosolic fractions (compare Figure 3K,L). The greatest increases in total AA-DNA adduct levels were observed in hepatic cytosols isolated from rats pre-treated with AAI/AAII mixture and incubated with AAI (10.9-fold; Figure 5C) and AAII (11.9-fold; Figure 6C) compared to cytosols isolated from control (untreated) rats. In contrast, total AA-DNA adduct levels were only 2-fold higher in renal cytosols isolated from rats pre-treated with AAI/AAII mixture relative to control (untreated) rats, either in incubation with AAI (Figure 5D) or AAII (Figure 6D). 

Pre-treatment of rats with AAI, AAII or AAI/AAII mixture also led to enhanced AA-DNA adduct formation (up to ~2-fold) in *ex vivo* hepatic or renal microsomal incubations with AAI (Figure 5A,B) and AAII (Figure 6A,B). For incubations with AAI or AAII and hepatic microsomes, the increases in total AA-DNA adduct levels corresponded to increased CYP1A enzyme activity levels (compare Figure 3A,C,E). For incubations with AAI or AAII and renal microsomes, no such association was found. 

## 3. Discussion

The extract of AA prepared from plants of the *Aristolochiaceae* family has been classified as carcinogenic to humans (group I) by the IARC [8]. It is responsible for two serious renal diseases, AAN and BEN, both of which are associated with the development of upper urothelial cancer [9,10,11,12,13,15,16,17]. Despite the known carcinogenic properties of AA [2,3,4,5,6,7], *Aristolochiaceae* species are still used in traditional herbal medicine, particularly in Asia, where potentially millions of people are exposed to the nephrotoxic and carcinogenic effects of AA [11,39,53,54,55,56,57,58,59]. While AAI, the major component of the plant extract, has been identified as a crucial factor for the development of UUC by inducing specific A:T→T:A transversion mutations in the DNA of patients suffering from AAN and BEN [4,13,38,40,42], the effects of the second major component, AAII, remain to better understood. Importantly, AA genotoxicity is mediated not only via AAI, but also via AAII, which also forms DNA adducts in urothelial tissue of AAN patients and in target organs of experimental animals [23,24,25,26,27,28,29,30,33,34]. Considering the fact that patients suffering from AAN or BEN or users of traditional herbal medicine are exposed to the natural mixture, which consists of both AAI and AAII, it is of high importance to understand how AAI and AAII contribute to AA-induced genotoxicity, how AAII can affect AAI metabolism and *vice versa*; however, no comprehensive study has yet been carried out to our knowledge. 

The aim of the present study was to investigate AAI and AAII metabolism, which are pathways of oxidative detoxification and reductive bioactivation, resulting in AA-DNA adduct formation in rats treated with AAI, AAII or AAI/AAII mixture. One key question of this study was how enzymes participating in AAI biotransformation are affected by co-exposure to AAII *in vivo*. Although previous studies showed AA-DNA adduct formation 24 h after administration, in the present study rats were sacrificed and analysed after 48 h in order to simultaneously measure and identify AA metabolites in urine [48]. 

The ability of both AAs to form covalent DNA adducts was studied using the ultra-sensitive ^32^P-postlabelling method, which was successfully employed previously and has been proven to be a powerful tool for understanding pathways of AA biotransformation. Our present study not only demonstrates the predictable formation of AA-DNA adducts in all rat organs tested, as described previously [23,24,32,36,44,60,61,62], but importantly the ability of AAII to enhance total AA-DNA adduct formation. The highest levels of AA-DNA adducts were observed in the renal tissue samples of rats, confirming the kidneys as the main target organs of genotoxicity, while the liver plays a role as the major biotransformation organ, particularly in the oxidative detoxification of AAI. Several studies have found that DNA adduct formation caused by AAI is higher than that by AAII [18,23,25,44]. These findings were supported by other data showing that AAII is a poorer substrate of biotransformation enzymes *in vitro* [26,27,30,45,49]; however, in the present study, total AA-DNA adduct levels in the liver were similar after treatment with AAI and AAII, while in the kidneys the total AA-DNA adduct levels were higher after treatment with AAII than AAI. This discrepancy might be explained by the various experimental models, treatment protocols and detection methods used or also by the different efficiency levels of enzymes participating in the detoxification and activation of AAI and AAII. Nevertheless, a study in *gpt* delta transgenic mice also showed that treatment with AAII induced higher levels of AA-DNA adducts in the kidneys than treatment with AAI [63]. More importantly, levels of dA-AAI adducts, which are known to be responsible for the induction of characteristic A:T→T:A transversion mutations, were elevated 3.6- and 4.5-fold in liver and kidney samples of rats treated with the AAI/AAII mixture compared to rats treated with AAI alone; thus, the presence of AAII might critically impact on the reductive activation of AAI to reactive cyclic acylnitrenium ions, thereby increasing AAI-DNA adduct formation. Moreover, these results confirm the findings of our study measuring urinary metabolites, which previously showed that AAII impacts AAI metabolism [48]. 

In the present study, AA-DNA adduct formation *in vivo* was overall in accordance with activity levels of AA metabolising enzymes determined in hepatic and renal microsomal and cytosolic fractions. AA can increase both the protein expression and enzyme activity level of cytosolic NQO1, the major enzyme reductively activating AA to reactive *N*-hydroxyaristolactam-nitrenium ions, resulting in AA-DNA adducts [15,21,61,62,64,65,66]. This was confirmed in the present study, further demonstrating that exposure to AAI/AAII mixture increased NQO1 activity by 1.5-fold in both hepatic and renal cytosols compared to cytosols isolated from rats treated with AAI alone; however, the underlying mechanism of NQO1 induction remains to be fully clarified. Some previous studies demonstrated that NQO1 induction caused by several chemicals, such as 2,3,7,8-tetrachlorodibenzo[1,4]dioxine, azodyes, butylated hydroquinones, Sudan I and III and polycyclic aromatic hydrocarbons, is closely associated with the effects of reactive oxygen species (ROS) [67,68,69,70,71,72]. It is believed that NQO1 is induced through the interactions of the transcription factor NRF2 with the antioxidant response elements in the promoter region of their genes. The transcription factor NRF2 is, under normal conditions, regulated by KEAP1, the protein targeting NRF2 for degradation; however, the presence of the above-mentioned chemicals, including ROS, causes the inactivation of KEAP1 protein, resulting in the accumulation of NRF2 in the nucleus. Consequently, higher levels of NQO1 are expressed [67,73,74,75]. Interestingly, ROS generation and oxidative damage to DNA were also described in some human cell lines after AAI exposure [76,77]; thus, the production of ROS caused by the treatment of rats with AAI (and AAII) might contribute to NQO1 induction.

The crucial role of NQO1 in AAI bioactivation *in vivo* and *in vitro* was described in previous studies [15,21,61,62,64,65,66]. Although the results of the present study indicate that NQO1 is responsible for the enhanced AA-DNA adduct formation *in vivo*, the increases in the levels of AA-DNA adducts formed in the kidneys and livers of the rats treated with AAI/AAII mixture compared to those treated with AAI alone does not fully correspond to the NQO1 enzyme activity detected in the renal and hepatic cytosols of these rats; thus, other mechanisms may contribute to the elevated levels of AA-DNA adducts in the liver and kidneys. This could include the ability of AAII to affect the oxidative detoxification of AAI. The oxidative metabolism of AAII has not been studied in detail, although this compound is present in high concentrations in some *Aristolochia* species and may even exceed the concentration of AAI [1,46,47]. Some studies assumed higher genotoxicity of AAII than AAI [63] and indicated that AAII induces more severe kidney and liver dysfunction [78]; however, other studies have demonstrated that AAII is not metabolised to AAIa *in vitro*, unlike AAI [49], and using the same rat model we previously showed that the major detoxification metabolites found in the urine were *N*-hydroxyaristolactam II and 7-hydroxyaristolactam II [48]. These findings indicate that AAII metabolism via activation is preferred to the oxidation pathway. Moreover, another study showed that AAII is capable of acting as a competitive inhibitor of AAI detoxification catalysed by CYP enzymes *in vitro*, with an inhibition constant (*K_i_*) of 11.3 μM [49]; hence, AAII might interfere in AAI detoxification, albeit it is itself not metabolised via these reactions. Subsequently, insufficient AAI detoxification might result in higher AAI concentrations in tissues, meaning more AAI is available for activation to cyclic acylnitrenium intermediates leading to AAI DNA adduct formation. Indeed, the inhibition of detoxification resulting in higher AAI concentrations available for reductive activation was found previously [61,62]. We suggest that this phenomenon might have occurred in the present study, i.e., the biotransformation of AAI resulting in increased AA-DNA adduct levels detected *in vivo*. According to previous results obtained during the first phase of our study, the levels of AAIa and aristolactam Ia, the main detoxification metabolites of AAI, found in the urine of rats treated with AAI/AAII mixture were ~2-3-fold decreased on the first day after administration compared to those treated with AAI alone [48].

The activation of AAI resulting in AA-DNA-adduct formation might also be modulated by the limited ability to detoxify AAI to AAIa [61,62]. Oxidative detoxification of AAI to the demethylated product AAIa has been investigated in detail in previous studies that identified CYP1A and CYP2C as the main enzymes catalysing these reactions in rats [20,52,79,80,81,82,83,84,85,86,87,88,89]. In the present study, enzymatic activity levels of CYP1A1/2 were determined using marker reactions, namely EROD for CYP1A, MROD for CYP1A2 and Sudan I oxidation for CYP1A1. The results demonstrated increased levels of CYP1A1/2 activity levels in the liver after treatment with AAI, AAII or AAI/AAII mixture. These findings suggest that there is increased capability of AAI detoxification in rat liver; however, although the most effective enzymes detoxifying AAI in rats and humans are CYP1A1 and CYP1A2 [79,81,82], the protein expression levels of these enzymes in the rat liver are very low (~2%) [90]. As such, the real contribution of the CYP1A subfamily to AAI detoxification in this organ is less significant than the contributions of other CYP enzymes. Specifically, enzymes of the CYP2C subfamily account for up to 55% of total CYP enzymes in rat liver [90] and are predominantly involved in AAI detoxification, particularly CYP2C11, which contributes up to 42% [52]. CYP2C11 is typically expressed in male rats [91] and represents approximately 50% of the CYP2C subfamily in the liver [92,93]; hence, the participation of CYP2C11 in the oxidative detoxification of AAI in rats is crucial for AAI metabolism and must be taken into consideration. Indeed, combined exposure of the rats to AAI and AAII (i.e., AAI/AAII mixture) resulted in a significant decrease (2-fold) in CYP2C11 enzyme activity compared to control (untreated) rats. Moreover, CYP2C11 activity decreased by 20% relative to the rats exposed to AAI alone. Furthermore, POR enzyme activity in hepatic microsomes did not change after treatment of rats with AAI/AAII mixture relative to control (untreated) rats, whereas it increased ~2-fold when rats were treated with AAI alone. POR is an essential component of the CYP monooxygenase system, where it supplies 2 electrons for CYP-mediated activation of the oxygen [94,95,96,97]. As such, decreased POR enzyme activity after treatment with AAI/AAII mixture might also contribute to less detoxification of AAI, thereby increasing AA-DNA adduct formation in rats.

On the other hand, both AAI and AAII induced enzyme activity levels of other detoxification enzymes in hepatic microsomes of treated rats, such as CYP2C6 and CYP1A. Although the contents of these enzymes are lower in the rat liver (10% CYP2C6 and 2% CYP1A), their contribution to AAI oxidation in rat liver is not negligible (both at ~17%) [52]. Here, the ability to oxidatively *O*-demethylate AAI to AAIa was demonstrated *in vitro* using hepatic and renal microsomes. Our results were in accordance with the elevated enzymatic activity levels of CYP1A1/2 and 2C6 determined in hepatic and renal microsomes isolated from rats treated with AAI or AAII. In hepatic microsomes, higher levels of AAIa were formed, confirming that the liver is the main organ responsible for AAI detoxification, as described previously [20,52,79,81,82,83,84,86,89]. Preliminary findings examining the hydroxylation of AAII to AAIa by hepatic and renal microsomes *in vitro* indicated that no hydroxylated (detoxification) product is formed (Stiborova et al., unpublished data). These findings are in agreement with previous studies [49] and our recent data showing that no AAIa was detectable in the urine of AAII-treated rats [48]. This indicates that AAII is metabolised specifically via the reduction pathway, ultimately leading to DNA adduct formation.

While the treatment with AAI or AAII alone resulted in similar increases in AAIa formation (~1.5-fold) in hepatic microsomes compared to control (untreated) rats, treatment with AAI/AAII mixture led to a greater increase (almost 2-fold). This indicates that despite the inhibition of CYP2C11 enzyme activity leading to a considerable decrease in AAI detoxification in the rat liver, treatment with AAI/AAII mixture positively affects CYP enzymes detoxifying AAI, particularly CYP1A1 and 1A2. Nevertheless, both CYP1A1 and 1A2 play a dual role in AA metabolism, since they are capable of both detoxifying and activating AAI. Previous studies have highlighted this phenomenon using genetically modified experimental animals (e.g., *Cyp1a* knockout mice), recombinant enzymes, specific inhibitors and inducers, *in silico* approaches and site-directed mutagenesis [52,60,61,79,82,86,87,98,99]. As such, we also tested the ability of microsomes to reductively activate AAI and AAII (i.e., catalyse AA-DNA adduct formation) *ex vivo*. Elevated levels of AA-DNA adducts were generated *ex vivo* in hepatic or renal microsomes when DNA was incubated with AAI or AAII. Increased AA-DNA adduct formation corresponded to the determined CYP enzyme activity, particularly in hepatic microsomes; however, while CYP1A1/2 mediated AA-DNA adduct formation in microsomes, in cytosols, AA-DNA adduct formation was mediated by NQO1. NQO1 enzyme activity was found to be elevated both in hepatic and renal cytosols isolated from rats pre-treated with AAI, AAII or AAI/AAII mixture.

**Figure 7:**
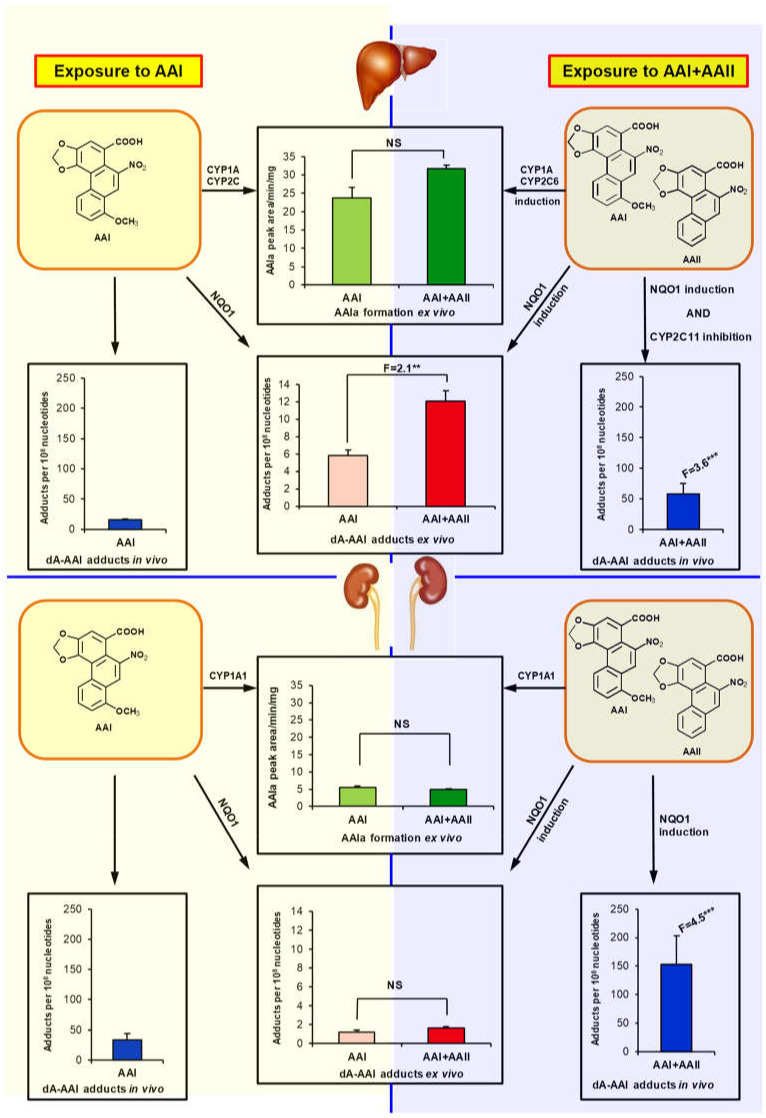
Schematic summary showing the effects of the presence of AAII on AAI-derived DNA adduct formation in the liver and kidneys in rats. Higher NQO1 induction in rats pre-treated with AAI/AAII mixture compared to rats pre-treated with AAI alone leads to increased AAI-derived DNA adduct formation (i.e., dA-AAI) *ex vivo* in rat hepatic cytosolic incubations with AAI; no change was observed in incubations with rat renal cytosols. AAIa formation was slightly increased in rat hepatic microsomes isolated from rats pre-treated with AAI/AAII mixture compared to rats pre-treated with AAI alone and incubated *ex vivo* with AAI. No differences in AAIa formation were observed in renal microsomes. Higher induction of NQO1 in livers and kidneys of rats treated with AAI/AAII mixture combined with hepatic CYP2C11 inhibition relative to rats treated with AAI alone leads to increased AAI-derived DNA adduct formation (i.e., dA-AAI) *in vivo*. Collectively, these results indicate that AAII present in the plant extract AA enhances the genotoxic properties of AAI (i.e., AAI DNA adduct formation). ‘F’ indicates fold increases in rats (pre)treated with AAI/AAII mixture compared to animals treated with AAI alone. Comparison was performed by *t*-test analysis: ** *p* < 0.01, *** *p* < 0.001. NS, not significant.

To summarise, our study has demonstrated increased AA-DNA adduct formation in the livers and kidneys of the rats treated with AAI/AAII mixture compared to rats treated with AAI or AAII alone. Enhanced AA-DNA adduct formation might be caused partially by both decreased AAI detoxification as a result of CYP2C11 inhibition during treatment with AAI/AAII mixture and by NQO1 induction, the key enzyme predominantly activating AA to DNA adducts. Moreover, our results indicate that AAII might act as an inhibitor of AAI detoxification *in vivo*, while AAII itself is not oxidatively detoxified by CYP enzymes to AAIa in rats. Consequently, higher amounts of AAI might remain in liver and kidney tissues, which can be reductively activated, resulting in enhanced AAI-DNA adduct formation. The complex cellular responses observed during AAI biotransformation in the presence of AAII are summarised in Figure 7. These results indicate that AAII present in the plant extract AA enhances the genotoxic properties of AAI (i.e., AAI DNA adduct formation). As patients suffering from AAN or BEN are exposed to the plant extract (i.e., AAI/AAII mixture), our findings are crucial to better understanding the host factors critical for AAN- and BEN-associated urothelial malignancy. Collectively, our results highlight that more attention should be given to AAII when mechanisms underlying AAI carcinogenesis of the plant extract AA are being examined.

## 4. Materials and Methods

### 4.1. Carcinogens

AAI (CAS number 313-67-7) and AAII (CAS number 475-80-9) were purified from the commercially available plant extract AA (Sigma, St Louis, MO, USA) by reverse-phase chromatography as reported previously [44]. The purity levels of AAI and AAII (as sodium salts) were checked by mass spectrometry (MS). High-resolution MS data were in agreement with data published previously [44].

### 4.2. Animal Treatment and Sample Preparation

The study was conducted as described previously [48] in accordance with the Regulations for the Care and Use of Laboratory Animals (419/2012, Ministry of Agriculture, Czech Republic), which is in compliance with the Declaration of Helsinki. Briefly, groups of male Wistar rats (~150–170 g, 5 weeks old; *n* = 3/group) were treated i.p. with a single dose of either 20 mg/kg body weight (bw) AAI, 20 mg/kg bw AAII or 20 mg/kg bw of AAI plus 20 mg/kg bw of AAII (AAI/AAII mixture). Animals in the control groups received the vehicle (corn oil) only. After administration, rats were housed in metabolic cages, which allowed the separated collection of urine and faeces. The results of these investigations were published previously [48]. On the second day after treatment, animals were sacrificed by cervical dislocation. Liver and kidney tissues were collected immediately, frozen in liquid nitrogen and stored at −80 °C. DNA samples from liver and kidney tissues used for DNA adduct analyses by ^32^P-postlabelling were isolated by phenol–chloroform extraction [31]. DNA quantity levels were assessed by UV-VIS spectrophotometry on a Carry 300 spectrophotometer (Varian, Palo Alto, CA, USA). Microsomes and cytosols were isolated from the rat tissues by procedures described previously [100,101]. Protein concentrations in the microsomal and cytosolic fractions were determined using the bicinchoninic acid protein assay with bovine serum albumin as a standard. Pooled microsomal and cytosolic samples (*n* = 3 rats/group) were used in further experiments. All microsomal and cytosolic samples were free of residual AAI, AAII or AAI/AAII metabolites, as determined by HPLC [79].

### 4.3. DNA Adduct Analysis by ^32^P-Postlabelling

The nuclease P1 enrichment version of ^32^P-postlabelling analysis with separation by thin-layer chromatography (TLC) on polyethylenimine–cellulose (PEI) plates was carried out as described previously [31,102]. TLC sheets were scanned using Instant Imager technology and DNA adduct levels (RAL, relative adduct labelling) were calculated as reported. AAI- and AAII-derived DNA adducts were identified using reference standards as described previously [31].

### 4.4. Enzyme Activity Assays

In hepatic and renal cytosols, NQO1 activity was measured using menadione (2-methyl-1,4-naphthoquinone) as a substrate; the assay was improved by the addition of cytochrome *c* and NQO1 activity, expressed as nmol cytochrome *c* reduced [79,99]. SULT1A1 enzyme activity was monitored by the formation of *p*-nitrophenol from a 5′-phosphoadenosine 3′-phosphosulfate (PAPS)-regenerating system [44,103,104]. Microsomal samples were characterised for specific CYP1A1/2 and 1A2 activity levels, namely ethoxyresorufin *O*-deethylation (EROD) for CYP1A1/2 and methoxyresorufin *O*-demethylation (MROD) for CYP1A2 [105]. CYP1A1 enzyme activity was also determined as the capability for Sudan I hydroxylation to 4′-hydroxy-, 6-hydroxy- and 4′,6-dihydroxy-Sudan I [106]. CYP enzyme activity can be modulated by cytochrome P450 oxidoreductase (POR), the electron donor to CYP enzymes. POR enzyme activity was determined using cytochrome *c* as a substrate [82,107]. The specific activity levels of CYP2C6 and 2C11 in hepatic microsomes only were characterised by their marker reaction, namely diclofenac 4′-hydroxylation and testosterone 16α-hydroxylation, respectively [108,109].

### 4.5. Microsomal Incubation to Study AAI Demethylation

Incubation mixtures contained 100 mM potassium phosphate buffer (pH 7.4), 1 mM NADPH, 0.5 mg rat hepatic or renal microsomal protein (microsomes of untreated (control) rats or rats treated with AAI, AAII or AAI/AAII mixture) and 10 µM AAI in a final volume of 500 μL, which were incubated at 37 °C for 10 min. AAI oxidation to AAIa was determined to be linear up to 25 min. Control incubations were carried out either (*i*) without microsomes, (*ii*) without NADPH or (*iii*) without AAI. AAI and its metabolite AAIa were separated by reverse-phase HPLC, identified by mass spectrometry and quantified as described previously [79]. Briefly, HPLC was carried out with a Nucleosil 100-5 C_18_, 250 × 4.0 mm, 5 mm (Macherey–Nagel) column, using a linear gradient of acetonitrile (20 to 60% acetonitrile in 55 min) in 100 mM triethylamonium acetate with a flow rate of 0.5 mL/minutes. A Dionex HPLC pump P580 with a UV/VIS UVD 170S/340S spectrophotometer detector set to 254 nm was used. Peaks were integrated with CHROMELEON™ 6.01 integrator. A peak eluting at a retention time (r.t.) of 24.329 min was identified as AAIa using mass spectroscopy analysis [79]. A typical HPLC chromatogram is shown in Figure 4C.

### 4.6. Cytosolic and Microsomal Formation of AAI- and AAII-Derived DNA Adducts

The de-aerated and nitrogen-purged cytosolic or microsomal incubation mixtures contained 50 mM Tris-HCl buffer (pH 7.4), 0.2% Tween 20, 1 mM NADPH, 0.5 mg rat hepatic or renal protein (i.e., either cytosols or microsomes isolated from untreated rats or rats treated with AAI, AAII or AAI/AAII mixture) and 0.5 mg calf thymus DNA (2 mM dNp), as well as 0.5 mM AAI, AAII or both in a final volume of 750 μL. Incubations with cytosols or microsomes were performed at 37 °C for 60 min. AAI- and AAII-derived DNA adduct formation was found to be linear up to 2 h [51,110]. Control incubations were performed either (*i*) without cytosol or microsomes, (*ii*) without NADPH, (*iii*) without DNA or (*iv*) without AAI or AAII. After extraction with ethyl acetate, DNA was isolated from the residual water phase by the phenol–chloroform extraction method, as described above. DNA adduct analyses using ^32^P-postlabelling were performed as described above.

### 4.7. Statistical Analyses

Statistical data analysis was performed used Student’s *t*-test. All *p*-values were two-tailed and considered significant at the 0.05 level.

## Data Availability

Not applicable.
